# Neurodevelopment and asymmetry of auditory-related responses to repetitive syllabic stimuli in preterm neonates based on frequency-domain analysis

**DOI:** 10.1038/s41598-019-47064-0

**Published:** 2019-07-23

**Authors:** Farveh Daneshvarfard, Hamid Abrishami Moghaddam, Ghislaine Dehaene-Lambertz, Guy Kongolo, Fabrice Wallois, Mahdi Mahmoudzadeh

**Affiliations:** 1 INSERM U1105, Université de Picardie, CURS, Amiens, France; 2 Faculty of Electrical and Computer Engineering, K.N. Toosi University of Technology, Tehran, Iran; 3 Cognitive Neuroimaging Unit, CEA DSV/I2BM, INSERM, CNRS, Université Paris-Sud, Université Paris-Saclay, NeuroSpin Center, 91191 Gif/Yvette, France; 4 INSERM U1105, Neonatal ICU, South University Hospital, Amiens, France; 5 INSERM U1105, Unit Exploration Fonctionnelles du Système Nerveux Pédiatrique, South University Hospital, Amiens, France

**Keywords:** Cognitive ageing, Paediatric research, Cognitive ageing, Paediatric research

## Abstract

Sensory development of the human brain begins prenatally, allowing cortical auditory responses to be recorded at an early age in preterm infants. Despite several studies focusing on the temporal characteristics of preterm infants’ cortical responses, few have been conducted on frequency analysis of these responses. In this study, we performed frequency and coherence analysis of preterm infants’ auditory responses to series of syllables and also investigated the functional brain asymmetry of preterm infants for the detection of the regularity of auditory stimuli. Cortical auditory evoked potentials (CAEPs) were recorded in 16 preterm infants with a mean recording age of 31.48 weeks gestational age (29.57–34.14 wGA) in response to a repetitive syllabic stimulus. Peak amplitudes of the frequency response at the target frequency and the first harmonic, as well as the phase coherence (PC) at the target frequency were extracted as age-dependent variables. A functional asymmetry coefficient was defined as a lateralization index for the amplitude of the target frequency at each electrode site. While the findings revealed a significant positive correlation between the mean amplitude at the target frequency vs. age (R2 = 0.263, p = 0.042), no significant correlation was observed for age-related changes of the mean amplitude at the first harmonic. A significant correlation was also observed between the mean PC and age (R2 = 0.318, p = 0.023). A right hemisphere lateralization over many channels was also generally observed. The results demonstrate that rightward lateralization for slow rate modulation, previously observed in adults, children and newborns, appears to be in place at a very young age, even in preterm infants.

## Introduction

Development of the auditory system is an intricate process beginning early in gestation^1^. Major structures of the ear, including the cochlea, develop between 23 and 25 weeks gestational age (wGA)^1–3^ and the capacity of the foetus to perceive and react to auditory inputs, related to brainstem network activation, emerges around 26 weeks of foetal life^4^. After 28 wGA, the thalamocortical auditory system is sufficiently mature to perceive complex sounds and discriminate between different speech phonemes^5–9^, corresponding to the beginning of language and speech development^10^. A critical period for neurosensory development of the auditory system starts around 25 wGA. During this period, the hair cells of the cochlea, the axons of the auditory nerve and the neurons of the temporal lobe in the auditory cortex are tuned to receive signals of specific frequencies and intensities^11^.

Cortical brain development can also be studied in preterm infants, who, as a result of intensive care, can survive when born after 28 wGA, and even 23 wGA^5^. Arousal behaviour confirms that the auditory system is already operative at this early age^12^ and threshold auditory sensitivity has been evaluated using auditory brainstem responses (ABRs)^13–16^. Hearing function rapidly improves from about 28 wGA to reach adult threshold sensitivity at term^17^. Cortical auditory evoked potentials (CAEPs) are recorded from 27 wGA^18^, with differences in wave amplitude and latency as a function of age^18,19^. Sound discrimination capacities, associated with mismatch responses, have been observed in response to changes of syllables and voices in healthy neonates tested ten weeks before term (28–32 wGA)^5–7^.

Auditory steady-state responses (ASSRs) is another methodological approach to study auditory development. ASSR measures the ability of the auditory network to fire synchronously with the rhythm (i.e., modulation rate) of an auditory stimulus^20^. ASSRs evoked by brief recurrent tones over a wide range of stimulus repetition rates have been assessed at different ages^21^. At high repetition rates, the magnitude of the responses at the trained frequencies was considerably lower in children than in adults. In infants aged 3–10 months, ASSRs in response to repetitive speech syllables revealed a systematic increase in the amplitude of the harmonics^22^. Recently in^5^, phase coherence analysis in 30 wGA preterm infants showed that immature neurons were able to follow a sequence of 4 syllables separated by 600 ms of silence, with reproducible topographies. Because of habituation, the amplitude of the evoked response decreased with repetition, with a weak, although significant phase-locking value across trials.

Although the auditory stimuli presented in^5^ does not correspond to a classical ASSR paradigm, regular repetition of the stimuli motivated us to study the ability of the immature auditory network to be entrained by the syllabic rhythmicity. As the frequency of stimulation is known, the same frequency can be studied in neural responses in order to obtain information on the ability of the immature network to follow the frequency of the auditory stimulation. This measure is less sensitive to background noise and analyses are reduced to the specific frequency of stimulation^21^. Variations of conductivity between the electrode and the scalp due to gel injection also do not affect the EEG frequency content, in contrast with its amplitude^23^. Furthermore, because the left and right hemispheres have different structural and functional maturational profiles^24^, and because a certain degree of hemispheric asymmetry of auditory responses has been described in adults^25^ that may affect the functional responses, we also analysed the lateralization of the responses.

## Methods

### Participants

EEG signals of 16 preterm infants with mean recording age of 31.48 wGA (29.57–34.14 weeks), recruited in our previous study^5^, were reanalyzed for the purposes of the present study. All infants had appropriate birth weight, size, and head circumference for their term age. The electroencephalogram was considered to be normal for gestational age. The infants were considered to be at low risk for brain damage on the basis of normal auditory and clinical neurological assessments. Written informed consent was provided by the infants’ parents and the study was approved by the Amiens University Hospital local ethics committee (CPP Nord-Ouest II) according to the guidelines of the Declaration of Helsinki of 1975 (ref ID-RCB 2008-A00728-47).

### Procedure design and stimulation

The stimuli consisted of fofur syllables (/ba/and/ga/, produced by male and female speakers) presented at a comfortable hearing level (≈70 dB) via speakers placed at the infant’s feet^5,6^. Syllables were matched for intonation, intensity, total duration (285 ms), prevoicing and voiced formant transition duration (40/45 ms). They were presented in series of 4 separated by 600 ms intervals. Five series separated by 1600 ms of silence constituted a block. Each block lasted 20 s and was followed by 40 s of silence. In each block, the repeated syllable was randomly chosen from among the 4 possible syllables. This presentation was applied in our previous study to evaluate syllabic discrimination in early preterm infants^5,6^. While in the standard trials, the same syllable was repeated four times, in deviant trials, the last syllable differed from the first three in voice or phonetic dimension. Although different from the classical ASSR paradigm, this regular presentation allowed to study the ability of the preterm brain to follow external ecological stimulations. Therefore, only the recorded responses corresponding to the standard trials, were used in this study for further analysis. Our previous results have shown that infants of this age are able to discriminate the two phonemes (/ba/vs./ga/) and the two voices (male vs. female) with distinct neural networks^5,6^.

### EEG recordings and preprocessing

The EEG signals were recorded using Ag/AgCl surface electrodes and a nasion reference. The sampling rate was 2048 Hz and the signals were amplified by A.N.Ts (Enschede, The Netherlands) and filtered at DC-50 Hz. The impedance of the electrodes was kept below 5 kΩ and the number of the electrodes (31–61) was determined according to the infant’s head circumference. A minimum of 31 electrodes were placed on the 10–20 points for all infants and additional electrodes were placed on intermediate positions according to the infant’s head circumference.

The recorded signals were band-pass filtered between 1 and 20 Hz and down-sampled to 512 Hz. They were segmented into epochs ([−0.5 + 2.7 s]) time-locked to the first syllable (S1) of the trial, providing 180 standard trials from each recording electrode for each subject. Trials were then baseline corrected to the 200 ms before the S1 onset. An automatic artifact-rejection procedure was applied as follows. Each trial was rejected if its absolute amplitude exceeded 50 µV or when a local amplitude jump between ten successive time-points exceeded 30 µV. The average number of 156 trials was remained for each infant and each electrode after the artifact exclusion.

### Frequency analysis

As the brain was exposed to a syllable every 600 ms, we expected a distinctive peak around 1.6 Hz and its harmonics. Temporal response corresponding to the time interval of the trials with the baseline removed ([0 + 2.7 s]), was transformed to the frequency domain using FFT. We, then, measured the peak amplitudes of the FFT spectrum at the target frequency and its first harmonic and tested the effect of age on this measurement. The amplitudes of the FFT spectrum at the target frequency and the first harmonic were extracted for each subject and each electrode and averaged across the 15 channels (Fz, F1, F2, F3, F4, C1, C2, CP1, CP2, Pz, P1, P2, Oz, O1 and O2) for all the subjects. These 15 channels are the most common channels showing activity between different subjects. Data analysis was performed by means of MATLAB R16 and the number of FFT points was adjusted at next power of 2 from length of signal.

### Phase coherence analysis

We applied phase coherence (PC) as a complementary frequential feature to study the preterms’ response to the auditory stimulation. Calculation of phase coherence^26–33^ requires segmentation of the CAEP into multiple subaverages. Subaverages are then transformed into the frequency domain using FFT. In the present study, sets of 10 trials were averaged, providing several independent subaverages from the total trials available for each electrode of each subject. Each averaged waveform was submitted to FFT spectral analysis. PC was determined by means of the following equation:

1 $$PC={[{(\frac{1}{n}\sum cos{\Phi }_{i})}^{2}+{(\frac{1}{n}\sum sin{\Phi }_{i})}^{2}]}^{1/2}$$

where *Φ*_*i*_ is the phase angle of the Fourier component of the ith subaverage, and n is the number of subaverages. PC ranges between 0 and 1, which is directly proportional to variability. It estimates the degree to which the phases at each frequency are dispersed or clustered. The more identical the average phases are, the more the PC value tends towards one. The PC value at the target frequency of the auditory response was tested for its age-dependency. We choose the PC value at the target frequency, as it constitutes the most significant peak in which the response is more closely tuned to the stimuli and at which the coherence is expected to be maximal.

### Lateralization index

A functional asymmetry coefficient was defined as a lateralization index, LI=(Right-Left)/(Right + Left), for the most prominent feature, the amplitude of the target syllabic frequency, at each electrode site and submitted to a *t*-test. The normalized value, Right/(Right + Left) or Left/(Right+Left), is less influenced by the intersubject variability related to the individual level of auditory maturation. Therefore, it provides a more sensitive measure than the original indices (Left or Right) for statistical comparison of right and left hemisphere activities. The number of repeated measures was corrected with False Discovery Rate (FDR) correction.

## Results

**Figure 1:**
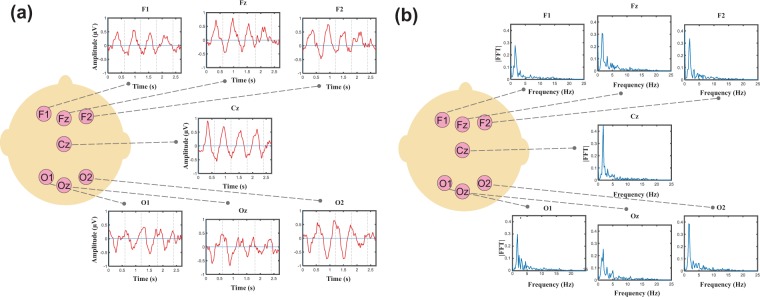
(**a**) Grand averages of the responses for several electrodes, (**b**) Fourier transforms of the grand averages. High frequencies are not illustrated as they contain values near to zero.

**Figure 2:**
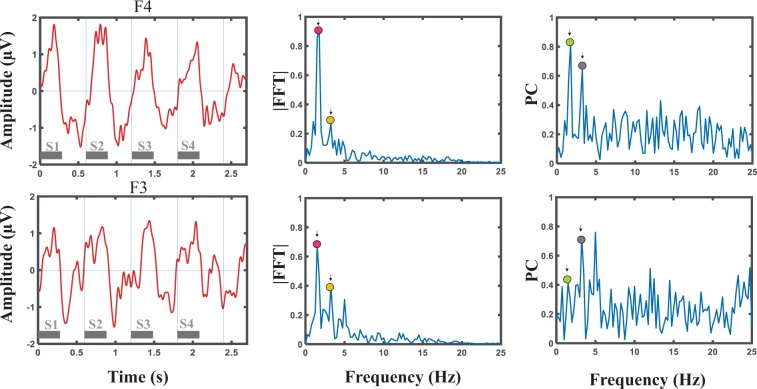
Temporal responses, FFT and PC spectra for an individual neonate (30 weeks and 4 days GA) at one right (F4, top row) and one left (F3, bottom row) electrode. The peak amplitudes corresponding to the target frequency and the first harmonic are marked in the FFT and PC spectra.

Grand averages of the responses are depicted in Fig. 1a for several electrodes. Fourier amplitudes of the grand averages are presented in Fig. 1b. In the previous study^5^, using the same stimuli and Global Field Power (GFP) analysis, the evoked responses to each syllable induced peaks with complex topography in the time domain. While the maximum positivity in the ERP is observed in frontal area, the maximum negativity is observed in posterior area, as described in^5^, suggesting two simultaneous dipoles located in the bilateral temporal areas. The temporal responses as well as the FFT and phase coherence spectra at one right (F4) and one left (F3) electrode are illustrated in Fig. 2 for an individual neonate (30 weeks and 4 days GA). As expected, distinctive peaks were observed at the target frequency and the first harmonic.

**Figure 3:**
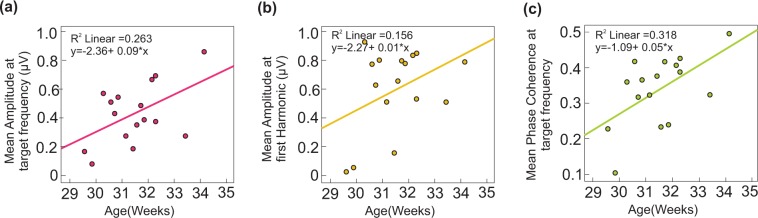
(**a**) Significant positive correlation between the mean amplitude at the target frequency across 15 channels and age, (**b**) Positive trend between the mean amplitude at the first harmonic across 15 channels and age, (**c**) Significant positive correlation between the mean PC at the target frequency across 15 channels and age.

A global increase of the amplitudes of the FFT spectrum at the target frequency and its harmonic was observed with increasing age (Fig. 3a,b), captured by a positive correlation between amplitude and age, significant at the target frequency (R^2^ = 0.263, p = 0.042), but not at the first harmonic (R^2^ = 0.156, p = 0.13). An age effect (R^2^ = 0.318, p = 0.023, Fig. 3c) was also observed for the phase coherence averaged across the 15 channels in each infant.

**Figure 4:**
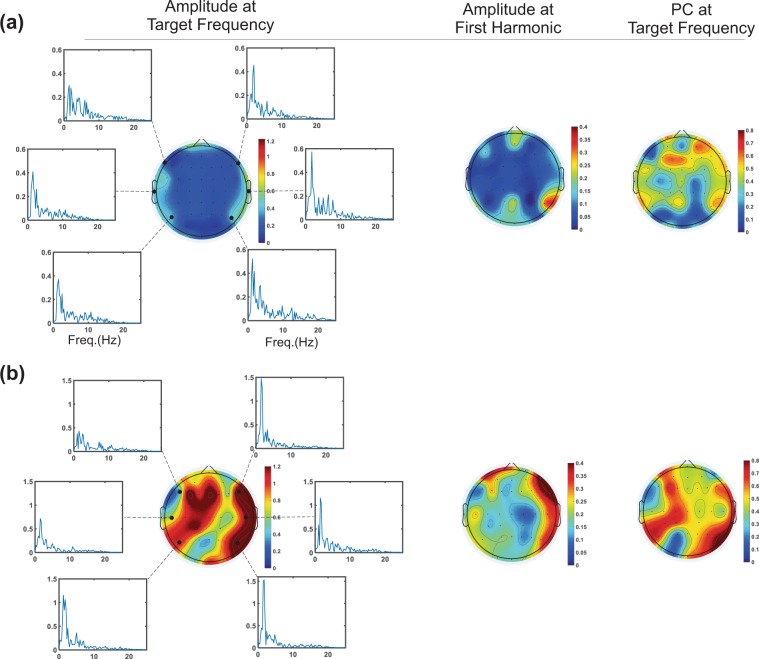
Topographic maps presenting the peak amplitude of the auditory responses at the target frequency, the first harmonic, and the PC at the target frequency for two neonates, at 31 weeks and 3 days GA (**a**) and 34 weeks and 1 day GA (**b**).

Topographic maps presenting the peak amplitude of the responses at the target frequency, the first harmonic and the PC at the target frequency are illustrated for two neonates, at 31 weeks and 3 days GA (Fig. 4a) and 34 weeks and 1 day GA (Fig. 4b). These topographic maps were created to provide the distribution of the extracted features all over the head. They exhibited an asymmetric pattern which motivated us to investigate asymmetry in more details. They also illustrated developmental changes between two subjects with different ages. However, developmental changes and asymmetry are more evident on the maps corresponding to the first frequency feature (first column).

**Figure 5:**
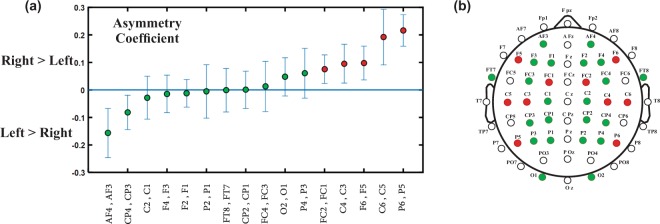
(**a**) Asymmetry coefficients calculated as the average of the LI values across the subjects for each pair of the available electrodes. The error bars indicate the standard error, (**b**) Locations of the available pairs of electrodes over the head. Electrodes with rightward biases are marked by red circles in both figures. The green circles show the channel pairs with no rightward bias. The white circles correspond to the channel pairs not investigated due to insufficiency of the available data.

Asymmetry coefficients were calculated for each pair of available electrodes by averaging the LI values across subjects (Fig. 5a). To obtain a fine-grained lateralization map, we considered all the possible pairs of electrodes (more than 15 common channels used for previous analysis) and performed one-tailed *t*-test analysis at each site as several studies have reported a rightward lateralization at early ages^6,34,35^. A robust rightward bias was observed for some of the auditory-involved areas marked in Fig. 5a,b by red circles (Corrected p-values: P6-P5: *p* < 0.001, C6-C5: *p* = 0.029, C4-C3: *p* = 0.045, F6-F5: *p* = 0.043, Fc2-Fc1: *p* = 0.043). The effect size of the differences between the means of paired channels was also calculated as the Cohen’s d (P6-P5: d = 2.080, C6-C5: 1.343, C4-C3: 0.779, F6-F5: 0.859, Fc2-Fc1: 0.836). Furthermore, we checked for the laterality in the opposite direction. Although Af4-Af3 (d = −1.012) and Cp4-Cp3 (d = −0.828) presented p-values less than 0.05, they showed no significant leftward lateralization after FDR correction. Finally, we did not observed variations of the lateralization index with age in the age-range considered in the present study.

## Discussion

Frequency and coherence analyses of CAEPs were performed in very-early preterm infants in order to investigate the temporal accuracy of the immature auditory network. This type of analysis, focused on a precise frequency band, is more robust to the background noise. ASSRs have been previously used to evaluate responses to low modulation rates, as present in speech^25,36,37^ and their hemispheric asymmetries^25,37–40^. The right hemisphere is thought to be sensitive to auditory modulations close to the speech syllabic rate^38–40^, whereas bilateral^38,40^ or left hemispheric^39^ specialization is reported for faster modulations. Although right asymmetry for slow modulations has been previously reported in adults^25^, children^41^, and newborns^42,43^, it was unclear whether some lateralization might already be observed in preterm infants. Our results suggest that a rightward bias is already in place in preterm infants.

### Age-related changes in auditory cortical responses

The human infant undergoes critical development of the auditory system during the period between 25 wGA to 5 or 6 months of age^11^. In the age range considered in our study (29.57 to 34.14 wGA), neurons are reaching their target locations and generate many connections within the cortical plate^44^. This neural synaptogenesis provides the basis for increased synchronization within a larger population of neurons, resulting in more clearly defined evoked potentials relatively to the background noise. This mechanism provides the structural background for the significant positive correlations, observed between age and the amplitude and PC value at the target frequency. The increase in phase coherence reveals that the neuronal response is more and more in line with the stimulus whereas the increase in amplitude might be related to the number of neurons and to the strength of their firing. Both features uncover the functional auditory improvement during the third trimester of pregnancy.

### Asymmetry analysis

In adults, the temporal/spectral acoustic properties of the auditory stimuli determine the relative hemispheric lateralization of their processing^44,45^. In general, broad-band auditory stimuli with rapid changes and temporal complexity are predominantly processed in the left hemisphere, while slowly changing narrow-band stimuli and spectral processing of sounds^44^ are essentially processed in the right hemisphere^46,47^. It has been hypothesized that each hemisphere is dominant for analysing modulations at different timescales and that phoneme-rate modulations lateralize to the left hemisphere, while rightward specialization is predominantly involved in low-frequency ASSRs and slower modulation rates such as syllabic-rate modulations^25^.

While previous works have focused exclusively on lateralization in adults and infants, only a few studies have investigated the asymmetry in preterm infants. Using near-infrared spectroscopy at the same preterm age as the current study, larger BOLD responses were recorded over the right frontal and temporal regions when preterms were processing the same syllables as here. Only the left posterior temporo-parietal region escaped this general pattern and displayed a faster and more sustained response than the right^6^. The infants included in the current study were selected from those recorded in^5^. The selection was based on the signal to noise ratio (SNR) in the frequency domain of the evoked auditory responses to syllable presentation. Three neonates were excluded from the analysis due to the low SNR.

Several other structural and functional studies reported a rightward asymmetry in preterm neonates’ development. The right Superior Temporal Sulcus (STS) is larger than the left one in preterm infants^48^ as in adults^49–51^ and some right sulci appear earlier than their left counterparts^52^. Resting state cerebral blood flow is larger^53^ and EEG power is higher in the right hemisphere relative to the left in premature neonates^54^. Syllables elicited larger right than left responses in premature infants and the discrimination responses to both a change of voice, and of phoneme induced a response in the right Inferior Frontal Gyrus^6^. The present study further shows that at a low-frequency rate, a rightward asymmetry both in terms of amplitude and of phase coherence is noticeable in many EEG channels, consistent with the results in adults^25^, children^39^, and newborns^40,41^. Thus, hemispheric asymmetries are a property of the developing human brain, already present at multiple structural and functional levels during gestation.

The asymmetry index did not change with age during the preterm period contrary to what has been reported after term for fast auditory modulations. Although at term, bilateral processing of fast auditory modulation has been reported in newborns^42,43^, hemispheric biases are changing with age and rate of modulation in a complex pattern^55,56^, probably influenced by maturation and experience^57^. Using a monaural stimulus at 4 Hz, Vanvooren *et al*.^37^ showed that right hemispheric specialization for processing syllable rate modulations appears to be mature in prereading children, at a very young age^37^.

Current findings, together with previous observations^5,6^, could have important implications for monitoring the emergence and normal development of specific brain functionalities in preterm infants. More specifically, frequency analysis is very suitable for studies in preterm and term neonates and can provide a useful approach to assess certain functional aspects of the capacity of auditory circuits underlying normal neurodevelopment.

## Conclusion

This study, using a frequency approach, demonstrates the ability of the preterm infant’s auditory network to process slow modulation with a rightward hemispheric lateralization. This ability is observed at a time when thalamocortical fibres invade the cortical plate, suggesting a strong temporal and spatial genetic fingerprint for auditory processing. The positive correlation between the extracted functional features and age suggests that this ability matures during the third trimester.
